# Data leaking scandal, risks, and financial consumption behaviors in online tourism platforms: The role of trust on college students and teachers

**DOI:** 10.3389/fpsyg.2022.968271

**Published:** 2022-08-03

**Authors:** Mingfen Feng, Bin Hu, Yaqi Tian, Huabing Wang

**Affiliations:** ^1^College of Economics and Management, Northeast Agricultural University, Harbin, China; ^2^School of Finance and Trade, Wenzhou Business College, Wenzhou, China; ^3^Department of Diplomacy and Foreign Affairs Management, China Foreign Affairs University, Beijing, China; ^4^School of Accounting, Guizhou University of Finance and Economics, Guiyang, China

**Keywords:** data leaking, financial consuming intention, defensive behaviors, online tourism platform, trust

## Abstract

Given the importance of data safety for psychology, the present study investigated the influence of data leaking scandal on campus customers’ financial consumption behaviors at intelligent tourism platforms in China, and explored the roles that individual characteristics play in this process by focusing on a set of participants from colleges. Data were collected through sending out an online questionnaire, where respondents were asked to finish a series of questions about their background information, their trust, future consuming intention, and defensive behaviors toward intelligent platforms. After they finished these questions, a short description about an online tourism platform leaking customers’ personal information was presented to the respondents, following which they were asked to report about their future consuming intentions and defensive behaviors again. In total, 236 participants of college students and teachers were recruited. Paired samples mean comparison showed that after the stimulus was presented, the respondents had a significant decrease in future financial consumption intention, and a significant increase in defensive behaviors toward online tourism platforms due to risks perceived. Multiple regression analysis was conducted subsequently to investigate individual characteristics that may account for part of the decrease (increase) in consuming intention (defensive behaviors). Results showed that, customers with higher level of trust and monthly income, as well as older customers, tend to experience higher level of decrease in consuming intention, and increase in defensive behaviors. These findings highlighted the importance of online tourism platforms guaranteeing data security of their customers.

## Introduction

In the era of business intelligence, data has been accepted as a new factor of production, and the correlation between personal data holding and business competitiveness has become more and more significant (Müller et al., 2018; Lee et al., 2020). The “big data” environment also intensifies the occurrence of data leakage. At present, mobile applications (APPs) have penetrated into social networking, video, e-commerce, travel and other fields, and the scale of data involved far exceeds that of traditional industries. In order to stimulate the potential commercial value of personal data, the phenomenon of excessive collection of personal data by APPs has repeatedly occurred, and the problem of how to use and whether to protect personal data by apps that excessively collect personal data is closely related to the personal rights and interests of users. In the past few years, the occurrence of information leakage of college students still cannot be eliminated, when serious information leakage will affect the mental health of college students. Zhou (2019) puts forward that as college students’ life practices become closely related to mobile applications, the daily arrangements of Chinese college students are almost full of them. However, every transaction means that we are exposed to risk, and the dynamics and fragmentation of the transmission of personal identity and financial information make leaks frequent. The psychological and mental health impact on students as a result of financial and private data breaches is thought to be significant and is most pronounced in developing countries (Ysseldyk et al., 2019).

In 2020, China’s Ministry of Industry and Information Technology punished 15 APPs for collecting personal information without permission, excessively claiming permission, and sharing it with third parties. Obviously, APP’s improper acquisition and inadequate protection of data result in massive data leakage, which seriously threatens the security of users’ personal information. The issue of online platforms leaking customers’ private data (e.g., credit card information) has attracted increasing attention, as it may cause serious consequences such as reduction in customers’ consumption intentions to continue visiting these platforms (see Baruh et al., 2017 for a meta-analytical review). Among which, leaking of personal data hold by online tourism platforms may be particularly concerning, since these data cover customers’ sensitive financial and personal information such as flight and hotel reservation and price (Cui et al., 2015). As a matter of fact, the scandal that one of the biggest tourism platforms in China leaked customers’ personal data has caused public panic about data security (Cui et al., 2015).

Nevertheless, previous studies rarely looked into how a data leaking scandal of online tourism platforms may affect customers’ financial consumption behaviors. Specific data development of current tourism platforms will be briefly discussed in the literature review. “Smart tourism” is an important development direction in China (Wang et al., 2021). At present, people in Chinese campus, such as college students, have largely relied on smart or intelligent travel platforms such as Ctrip to make travel arrangements. In the process of incorporating business intelligence into intelligent travel platforms, there are often improper push algorithms and data protection methods. In addition, intelligent tourism platforms often need to obtain users’ multiple privacy information and financial information. It has been reported that a large number of users’ bank card information (including cardholder’s name and ID card, bank card number, CVV code) was leaked on a famous smart tourism platform due to no protection in secure payment log. The scandal caused widespread social anxiety and flooded banks with inquiries. It is worth noting that college students are one of the main groups of victims due to their low awareness of prevention. These incidents pose a great threat to students’ spiritual and life safety. The present study therefore aimed to investigate this issue and also explored the role that individual characteristic (e.g., trust toward the platform) play in this process.

## Literature review

The diversified functions and convenience offered by the online tourism platforms makes it a priority for most potential customers planning to travel to get more help from the Internet when buying airline tickets, booking accommodation or researching attractions (Qinghao, 2022; Yuan et al., 2022). Therefore, Song and Liu (2017) points out that enterprises in the online tourism industry are increasingly using “big data” to generate new methods of decision-making, opportunities, and overall performance. This is reflected in a wide range of information technology tools that generally connect disparate information from different systems to each other to improve decision making (Niu et al., 2021). For example, Kim et al. (2021) points that the current big data technology and the support provided by Record Data can reduce the load bearing pressure and advance preparation of scenic spots by understanding the user’s preference for travel time. However, Höpken et al. (2020) points out that these original data are often processed by various clustering technologies through thresholds. In some cases, the data also involves photos and GPS tags to identify points people are interested in and learn about users’ preferences. This means that information about latitude and longitude locations, as well as personal privacy, is at risk if not kept properly, despite the best intentions of such data (Vu et al., 2018). In addition, Kovács et al. (2021) also mentions that similar data acquisition approaches include travel activity records using mobile social media data. Data created in certain social media platforms about check-ins can be analyzed to identify travel patterns of visitors (Hasnat and Hasan, 2018). Available data generally contains a wealth of information about location, activities, and time spent. Therefore, by reconstructing site registration data, practitioners can analyze visitor movements (Li et al., 2019). Some scholars concern about the privacy risks on personal mobility (Masseno and Santos, 2018), however, it hasn’t raised much attention.

This paper argues that the financial risks of tourism platforms are underestimated in China. At present, with the continuous development of Internet and communication technology in China, the way of tourism information processing and transaction has changed from traditional face-to-face to electronic, a large number of financial traces related to tourism transactions therefore have been left. Shrestha et al. (2020) points out that these financial traces are reflected in a variety of tourism information, such as planning, information search and booking before a trip, and booking and post-trip experience sharing. In addition, it is worth mentioning that Marzouk (2022) added that the process of these financial activities is also accompanied by recommendations, and the information of photo uploading and other social media interactive activities should also be paid attention to. Volo (2020) points out that for online tourism platforms, these large, unstructured and complex electronic traces, or “tourism big data,” constitute the basis for mining user behaviors. Qiu and Qi (2020) believes that through data integration and analysis, hidden patterns and mutual relations in the tourism field can be found. For example, Xie et al. (2021) used big data analysis to reveal the behavioral economic model of tourism consumption. Jia et al. (2022) created a financial platform of smart tourism based on big data platform, and proposes the construction mode and path to maximize the benefits of “smart tourism.” However, the risk of financial information disclosure and the risk of privacy disclosure mentioned above have been neglected in the current research. Pencarelli (2020) points out that with the popularity of the Internet, more and more citizens have changed their trading and consumption habits, from customizing travel products in travel agencies to more inclined to trade online. At present in China, tourism complaints easily become hot spots on the Internet during holidays, among which young people, especially college students, are one of the victims. However, there is a lack of research on these phenomena in China. But it is not limited to China, there are similar situations in western countries. Travel company Carnival Corporation, for example, had a data leakage. Hackers had accessed the travel company’s employees’ electronic accounts without authorization, obtaining private data such as names, addresses, social security numbers, passports, driver’s licenses, and health information, as well as financial data such as credit cards and financial accounts. Considering that there is still a lack of research on this aspect, this paper conducts a pilot discussion on the impacts of data leakage about tourism platform.

## Methods

### Procedure and participants

An online survey questionnaire was prepared and conducted in Wenjuanxing^1^ which is a Chinese popular survey platform. Finally, 236 participants from colleges in Heilongjiang and Guizhou Provinces were recruited. This sample size is decided beforehand by using G*power, that the sample size to detect an effect of 0.2 with 90% power in *t*-tests is 207. The sample was balanced in gender (104 male, 44%), was diverse in age, with participant from age groups (1) under 19 (*N* = 5), (2) between 19 and 30 (*N* = 90), (3) between 30 and 40 (*N* = 61), (4) between 40 and 50 (*N* = 57), and (5) above 50 (*N* = *23*) years old. The sample also includes participants with different levels of monthly income, such as (1) below 3,000 (*N* = 33), (2) between 3,000 and 6,000 (*N* = 70), (3) between 6,000 and 9,000 (*N* = 70), (4) between 9,000 and 12,000 (*N* = 34), (5) between 12,000 and 15,000 (*N* = 18), and (6) above 15,000 yuan (*N* = 11). In the questionnaire, the respondents were firstly asked to finish questions about their demographic information, and several questions about their trust toward online tourism platforms, intention to continue consuming in online tourism platforms, and defensive behaviors due to perceived risks toward online tourism platforms. After finishing these questions, the respondents were presented a description about an online tourism platform leaking customers’ personal data (e.g., name and credit card information). After this stimulus was presented, the respondents were asked to answer questions about their future intention and defensive behaviors toward online tourism platforms again. Such process design was inspired by Chen and Zhang (2021) and Zhang et al. (2022).

### Measurements

Customers’ *Future Consuming Intention* was measured with five items tapping customers’ willingness to use tourism platforms in the future, such as, *In the future, I am willing to continue consuming in online tourism platforms*. These items are adapted from Questionnaire measuring *Customers’ Promotion-focused Behavior* from Lwin et al. (2015), and the reliability and validity of this questionnaire have been proved in previous research (Lwin et al., 2015). These items also showed high reliability (Cronbach’s alpha = 0.94) and construct validity (standardized factor loadings ranging from 0.85 to 0.90) in the present study. Customers’ *Defensive Behaviors* toward online tourism platforms was measured by five items reflecting their requests and actions to prevent tourism platforms from getting further contact with them, such as, *In the future, I will ask online tourism platforms to stop using my personal information.* These items were adapted from the questionnaire measuring *Customers’ Prevention-focused Behaviors* by Lwin et al. (2015). The original questionnaire showed adequate statistical properties (Lwin et al., 2015), and in the present study, these items also showed high reliability (Cronbach’s alpha = 0.95) and construct validity (factor loadings ranging from 0.87 to 0.90). The respondents’ *Trust toward Online Tourism Platforms* was measured with five items about their positive beliefs about the reputation of the tourism platform, such as *I believe online tourism platforms are honest and trustworthy*. These items were developed based on the questionnaire measuring customers’ *Trust in Online Retailing* from Mukherjee and Nath (2007). One item is reversely worded and was recoded so that a higher score on this scale reflecting customers’ higher level of trust on tourism platforms. The original questionnaire had good reliability and validity in previous research (Mukherjee and Nath, 2007). These items also showed high reliability (Cronbach’s alpha = 0.94) and construct validity (factor loadings ranging from 0.85 to 0.90) in our sample. For all the items, the respondents needed to indicate to what extent they agree with the statements on a 5-point scale, from one (*No, I totally disagree)* to five *(Yes, I totally agree)*. Therefore, a higher score on the construct reflect a higher level of the measured traits.

### Analysis

Data were analysis in SPSS 25. As respondents were asked to finish all the questions, no missing values was present in our data. To investigate the influence of the data leaking scandal on customers’ consuming behaviors, we conducted paired sample *t*-tests, to compare the mean scores on Future Consuming Intention and Defensive Behaviors before and after the stimulus was presented. Cohen’s d was reported to indicate whether a significant effect was small (> 0.2), medium (> 0.2 and < 0.5), or large (> 0.8; Cohen, 1988). To further investigate the roles of customers’ individual characteristics in shaping their responses to the stimuli, multiple regression analysis was used, taking customers’ trust toward tourism platforms, gender, age, and monthly income as independent variables. The difference score in Future Consuming Intention (Defensive Behaviors) before and after the stimuli was calculated and taken as the dependent variables. Regression coefficients were all standardized to indicate whether significant effects were small (> 0.1), medium (> 0.1 and < 0.3), or large (> 0.5; Cohen, 1988). Based on the theoretical model proposed by Lwin et al. (2015), we developed the models as follows:


Consuming⁢behaviors

=Trust+Age+Gender+Monthly⁢Income+error,

where consuming behaviors refers to customers’ future consuming intention or defensive behaviors toward online tourism platforms.

## Results

Descriptive statistics and correlations between studied variables are present in Table 1. As it can be seen, there was a drop in the mean scores in Future Consuming Intention before and after presenting the stimulus, and the two scores were moderately correlated (*r* = 0.33, *p* < 0.01). On the contrary, there was an increase in the mean scores of Defensive Behavior before and after the stimulus is presented, also with a moderate correlation between the two scores (*r* = 0.33, *p* < 0.01).

**TABLE 1:** Descriptive statistics and correlations between studied variables.

	Mean (*SD*)	1	2	3	4	5	6	7	8
**Dependent variables**									
1. Future consuming intention T1	3.83 (1.09)	1.00							
2. Defensive behavior T1	2.12 (1.11)	−0.95**	1.00						
3. Future consuming Intention T2	2.46 (1.36)	0.33**	−0.33**	1.00					
4. Defensive behavior T2	3.92 (1.10)	−0.32**	0.33**	−0.96**	1.00				
**Individual characteristic**									
5. Trust		0.95**	−0.94**	0.34**	−0.33**	1.00			
6. Gender	−	0.01	0.02	–0.08	0.01	0.02	1.00		
7. Age	−	0.04	–0.06	−0.16*	0.13*	–0.01	0.04	1.00	
8. Monthly income	−	0.01	–0.03	−0.23**	0.17**	–0.02	0.07	0.09	1.00

As Gender (0 = male, 1 = female) is a dummy variable, and Age and Monthly Income are ordinal variables, spearman correlations are presented for these variables.

*p < 0.05, **p < 0.01.

Results of mean comparisons and regression analysis are presented in Table 2. After the data leaking scandal was presented, the respondents’ Future Consuming Intention decreased significantly (Mean difference = 1.37, *p* < 0.001), with a larger effect size (Cohen’s *d* = 0.96). On the contrary, the customers’ Defensive Behavior increased significantly after the stimuli was presented (Mean difference = 1.29, *p* < 0.001), with a large effect (Cohen’s *d* = 0.91).

**TABLE 2:** Results of mean comparisons and regression analysis.

	Paired samples mean comparisons					
	Future consuming intention			Defensive behavior		
	Before(T1)	After (T2)	*T (df)*	Before (T1)	After (T2)	*T (df)*
	3.83	2.46	14.82 (235)***	2.12	3.41	–13.94 (235)***

	**Regression analysis**					
	**Decrease in intention (T1-T2)**			**Increase in defensive behaviors (T2-T1)**		
	**Beta**	**S.E**	***P***	**Beta**	**S.E**	***P***

Trust	0.39***	0.75	<0.001	0.43***	0.07	<0.001
Gender	0.06	0.17	0.285	0.03	0.16	0.645
Age	0.13*	0.08	0.026	0.12*	0.08	0.037
Monthly Income	0.23***	0.06	<0.001	0.20***	0.06	<0.001
*R* ^2^	23.6%			24.3%		

*p < 0.05, ***p < 0.001.

To further investigate factors affecting the decrease in customers’ Future Consuming Intention and the increase in their Defensive Behavior, the difference scores were made by subtracting lower scores from higher scores, and were included in multiple regressions as dependent variables. The results showed that customers Trust toward tourism platforms had significantly positive effects on the decrease in Future Consuming Intention (Beta = 0.39, *p* < 0.001), and the increase in Defensive Behavior (Beta = 0.43, *p* < 0.001). Customers’ age also had significantly positive effects on the decrease in Future Consuming Intention (Beta = 0.13, *p* = 0.026), and the increase in Defensive Behavior (Beta = 0.12, *p* = 0.037), indicating that older customers are more tended to decrease their intentions and increase their defensive behaviors after knowing the scandal. In addition, customers’ Monthly Income had significantly positive effects on the decrease in Future Consuming Intention (Beta = 0.23, *p* < 0.001), and the increase in Defensive Behavior (Beta = 0.20, *p* < 0.001), indicating that customers with higher income are more likely to reduce their intention and take defensive actions toward data leaking. These models explained 23.6% of the variances in Decrease in Consuming Intention, and 24.3% of the variances in Increase in Defensive Behaviors.

## Discussion

The present study investigated the influence of a data leaking scandal on campus people’s consuming behaviors at online tourism platforms in China. Two major findings came out of the present study. First, customers in campus reduced their intentions to continue using online tourism platforms and increased defensive attempts toward these platforms after a stimulus about a data leaking scandal was shown. Second, in the process of decreasing intentions and increasing defensive behaviors, campus customers’ individual characteristics such as trust toward tourism platforms play important roles.

The decrease in future consuming intentions and increase in defensive behaviors confirmed the seriousness of the negative effect of data leaking scandals on tourism platforms. This finding is in line with findings from previous studies that when customers feel concerned about security in online purchasing, their purchasing loyalty tends to decline (Cui et al., 2015). Considering that presenting a vignette can already produce such large effects, this study highlights the importance of data security to keep customers. Therefore, online tourism platforms should pay close attention to prevent data leaking, such as developing a detailed data security working manual (Liu et al., 2018). Otherwise, they may face severe credit crisis and substantial losses of profits.

In addition, the present study found that customers with higher trust toward tourism platforms tend to act more strongly toward the data leaking scandal. This finding confirms the argument made by Yun et al. (2014) that some individual characteristics may moderate the relationships between privacy risk and customers’ willingness to transact or provide personal information. A plausible reason is that customers with a higher trust may feel more offended and betrayed, and as a result, they would feel more disappointed about tourism platforms and take more harsh actions. This result indicates that, online tourism platforms may need to take good care of loyal customers that are more attached to their services. In face of data leaking, these customers may take more intense reactions and therefore may bring dramatic losses to online tourism platforms. Moreover, customers with higher income also tend to reduce their intentions and conduct defensive behaviors to a higher degree. This might be that customers with higher incomes often care more about their privacy, such as information about their credit card. This finding again warrants online tourism platforms, as customers with higher income usually tend to spend more and therefore losing this group of customers brings more economic losses. Similarly, older customers are more tempered to reduce their intentions and take defensive actions after reading about the data leaking scandal. A possible explanation is that older customers are often more conservative about online shopping, and therefore tend to refrain from consuming online once they are informed of the risk of data leaking. Taken together, these findings address an important issue that some groups of people may have stronger opinions about data leaking and therefore online tourism platforms may particularly take care of data security to win their trust.

## Conclusion

Through investigating Chinese college students and teachers’ reactions toward a data leaking scandal of an online tourism platform, the present study found that when informed of the potential risk of data leaking, customers tend to decrease their intentions to continue consuming in online tourism platforms, and increase their defensive behaviors toward these platforms, such as disallowing these platforms to use their personal information. Furthermore, individual characteristics play a role in shaping their reactions toward the data leaking scandal. College students and teachers with higher trust toward online tourism platforms, as well as older participants and participants with higher monthly income, tend to reduce future consuming intentions to a higher degree, and display higher level of defensive behaviors. Altogether, these findings address an important issue that data leaking can cause seriously negative effects to online tourism platforms. It is also found people on campus are one of the most vulnerable groups, especially college students. Given the stress of past data breaches, the present study advocate tourism platforms should think highly of data safety. This especially applies to specific groups of customers, as they tend to react even more strongly toward data leaking issues.

The present study is one of the first few studies to explore the influence of data leaking on campus customers’ behaviors at online tourism platforms, and this study shed light on the importance of promoting data security in campus. However, several limitations still need to be accounted and future research may further explore related issues based on our findings. First, we examined the influence of a data leaking scandal by presenting a stimulus to campus customers and mainly focused on campus customers’ instant reactions toward the information, and were not able to investigate the long-term effect of a data leaking scandal. Future research may therefore conduct longitudinal study to track how customers may react to a data leaking scandal in a long run. Second, although we already included customers’ individual characteristics such as trust toward tourism platforms, some other interesting characteristics were not discussed in the present study (e.g., customers’ privacy concern), future research may therefore include these characteristics as well and discuss their roles in shaping customers’ reactions toward a data leaking scandal.

## Data availability statement

The original contributions presented in the study are included in the article/supplementary material, further inquiries can be directed to the corresponding author/s.

## Author contributions

MF and BH performed material preparation, data collection, and analysis. MF and HW wrote the first draft of the manuscript. YT drafted and edited the revised manuscript. All authors read and approved the final manuscript and contributed to the study conception and design and all authors commented on previous versions of the manuscript.

## Footnotes

1
www.wjx.cn
